# Lymphangiogenesis in Oral Squamous Cell Carcinoma: Correlation with VEGF-C Expression and Lymph Node Metastasis

**DOI:** 10.1155/2017/7285656

**Published:** 2017-06-07

**Authors:** Manar A. Abdul-Aziz, Amina K. Amin, Dalia H. El-Rouby, Olfat G. Shaker

**Affiliations:** ^1^Department of Oral and Maxillofacial Pathology, Faculty of Oral & Dental Medicine, Cairo University, Giza, Egypt; ^2^Department of Medical Biochemistry and Molecular Biology, Faculty of Medicine, Cairo University, Giza, Egypt

## Abstract

**Background:**

Oral squamous cell carcinoma (OSCC) is the most common oral malignancy that preferentially spreads to the cervical lymph node which, when involved, complicates the anticancer therapy and threatens the patient life. It was suggested that lymph node metastasis may be facilitated by lymphangiogenesis. VEGF-C is one of the most important lymphangiogenic inducers that promotes the lymphatic vessels growth and supports the survival of adult lymphatic endothelial cells.

**Methods:**

Lymphatic vessels density (LVD) and LV morphometry were digitally evaluated using D2-40. The expression of VEGF-C was also assessed using immunohistochemistry and real-time polymerase chain reaction in 6 normal oral mucosa cases and 72 cases of OSCC. The correlation between LVD and LV morphometry, VEGF-C, and lymph node metastasis was statistically assessed.

**Results:**

A positive cytoplasmic expression of VEGF-C was detected in both epithelial and connective tissue cells in 97% of OSCC, while all normal tissues reacted negatively. A greater expression of VEGF-C was associated with larger and more dilated LV and lymph node metastasis but not with LVD.

**Conclusion:**

VEGF-C is actively involved in the invasion and metastasis of OSCC via inducing morphological changes in LV. VEGF-C may be a promising target for anticancer therapy.

## 1. Introduction

Oral cancer has occupied the sixth position in the cancer incidence ranking worldwide [1, 2] with OSCC representing 80–90% of all oral malignancies [3, 4]. Despite advances in anticancer therapy, the prognosis remains unfavorable and 50% of patients die from this disease. This is due to lack of effective diagnostic and prognostic methods which can guide and optimize appropriate treatment strategies at early stages [5]. Most deaths from cancer result from progressive growth and metastasis that resist the current therapies [6].

Several studies had graded OSCC into well, moderately, and poorly differentiated lesions and suggested the positive correlation between the lesser histological differentiation and the poorer prognosis [7]. However, the histological grading has not been closely related to the disease outcome as the presence of metastasis was not necessarily associated with the lesions of poorer differentiation [3].

OSCC, like the most of epithelial malignancies, preferentially spreads through lymphatic vessels. In fact, the spread to regional lymph nodes (LN) is an early event in systemic dissemination and, therefore, cervical node metastasis is widely accepted as one of the major prognostic factors in patients with OSCC [3, 8].

In the majority of the clinical studies, a significant correlation has been observed between LVD and lymph node and organ metastasis [9, 10] and suggested that LN metastasis is preceded by lymphangiogenesis (development of new lymphatic vessels) within or surrounding the tumor tissue [11, 12].

However, the area of lymphangiogenesis was not adequately assessed in carcinogenesis in early studies and, little was known about the mechanism of lymphangiogenesis and lymph node metastasis [6, 13]. This was because of the lack of specific markers that can discriminate the lymphatic endothelium from blood capillaries [10].

Recently, the discovery of a number of specific lymphatic markers has allowed novel insight into how the lymphatic vessels can affect the tumor progression and patient prognosis [8].

This set of markers comprises VEGFR-3 (Flt-4), the product of the* prospero*-related homeobox gene-1 (Prox-1), Lyve-1 [14], and the more specific marker, podoplanin (D2-40) [15].

However, the functional role of newly formed intratumoral lymphatic vessels is still debatable [11]. This is because the tumor-associated lymphangiogenesis is only present in some regions [16] and due to the late occurrence of lymphangiogenesis in comparison with the spread of tumor cells to regional lymph nodes [17].

Furthermore, some cases of breast tumors failed to induce lymphangiogenesis and lymphatic metastasis occurred only through the preexisting vessels [18], while, in other lesions of OSCC, LVD tended to decrease with their progression and metastasis [19].

Therefore, the assessment of lymphangiogenic markers may be more helpful as a prognostic indicator, compared to the lymphatic endothelial cell markers [19].

One of the most important lymphangiogenic inducers is vascular endothelial growth factor C (VEGF-C) which acts via activation of vascular endothelial growth factor receptor 3 (VEGFR-3) expressed by lymphatic endothelium. Such activation results in growth of lymphatic vessels and promotes proliferation, migration, and survival of adult lymphatic endothelial cells [9].

Overexpression of VEGF-C has been demonstrated in many human cancers and revealed that the expression of VEGF-C actively induces tumor-associated lymphangiogenesis, leading to lymphatic invasion, lymph node and distant metastasis, and subsequently poor patient survival [8, 20].

In OSCC, Watanabe et al. [19] reported that VEGF-C was significantly more likely to cause cervical lymph node metastasis and the increased positivity was associated with larger lymph nodes. However, in other studies that correlated the expression of VEGF-C by tumor cells with the lymph node status, the results were variably assessed and controversial findings were reported [9, 10, 21–23].

In the present study, we seek to investigate the expression of VEGF-C in OSCC and to focus on the correlation between its expression and LVD, LV morphometry, and lymph node metastasis.

## 2. Material and Methods

An ethical approval on the study design was obtained from Research Ethics Committee, Faculty of Oral and Dental Medicine, Cairo University.

### 2.1. Tissue Specimens

**Figure 1:**
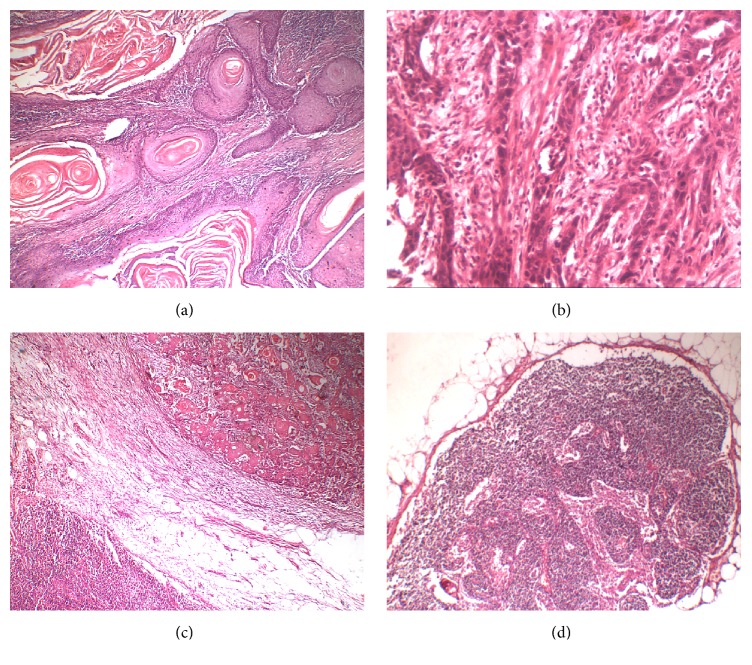
Photomicrographs of H&E stained sections showing different grades of OSCC ((a) ×40), ((b) ×100) and OSCC associated positive ((c) ×40) and negative ((d) ×40) lymph nodes.

A total of 72 formalin-fixed paraffin-embedded specimens of OSCC, divided into 36 positive and 36 negative lymph node lesions, were involved in this study (Table 1). The status of lymph node was determined for each case by histopathological examination of nodes removed with neck dissection (Figure 1). In addition, 6 normal mucosal tissues were also examined.

The paraffin blocks were retrieved from Faculty of Oral and Dental Medicine, Faculty of Medicine, Cancer National Institute, Cairo University, and Faculty of Dental Medicine, Alexandria University.

### 2.2. Immunohistochemical Staining Procedure

Immunohistochemistry was carried out using mouse monoclonal antihuman D2-40 antibody (Prediluted, Biocare Medical, USA), goat polyclonal anti-human VEGF-C antibody (5 *μ*g/mL, R&D system, UK), and universal detection kit (R&D system, UK).

The four *μ*m thick paraffin-embedded tissue sections were dewaxed in xylene, immersed in descending concentrations of alcohol, and then microwaved in antigen retrieval solution at 100°C for 10 minutes. To block nonspecific interactions, tissue sections were incubated in serum blocking reagent followed by avidin-biotin blocking reagent for 15 minutes each. The slides were incubated with primary antibody for 60 minutes in the humidity chamber at room temperature. Then, the slides were incubated with biotinylated secondary antibody, followed by incubation with streptavidin-horseradish peroxidase conjugate for 30 minutes. The peroxidase activity was made visible with diaminobenzidine (DAB). Counterstaining was done with Mayer's hematoxylin.

### 2.3. Assessment of Immunostaining

The ordinary light microscope was used to detect and localize the positive reaction within the tissues.

#### 2.3.1. Assessment of VEGF-C Expression

The immunostained sections were examined using Leica Qwin 500 analyzer computer system (Germany). The immunoreactivity of VEGF-C was measured as optical density and area and area percent in a standard measuring frame of area 38883.69 micrometer^2^ per 10 fields using magnification (×200) by light microscopy transferred to the screen. Small sections with overlapping fields or those damaged by immunohistochemical procedure were excluded from the assessment. 30 cases in each group of OSCC were included.

The area and area percent of VEGF-C expression was also assessed in epithelial cells and cancer associated stromal cells separately.

#### 2.3.2. Quantification of LVD

Using D2-40, the density of lymphatic vessels (LVD) was evaluated according to the methods described by Audet et al. [24].

First, all slides were screened using a low-magnification objective lens to identify the areas that contained the highest number of positively stained vessels (hot spots). Then, the number of vessels was counted by 2 observers in three hot spots using (×200) magnification lens and the mean of the vessel counts per field was recorded.

#### 2.3.3. Morphometric Analysis of LV

After identification of lymphatic vessels by podoplanin immunostaining, each lymphatic vessel was manually selected using the cursor and the computer system automatically calculated surface area (*μ*m^2^) and circularity (degree of roundness) of the lymphatic vessels. A value of 1.0 indicates a perfect circle.

### 2.4. RT-PCR Procedure

This procedure was conducted on 30 cases of OSCC, divided into 15 positive and 15 negative node lesions, in addition to 6 normal tissue specimens as a control group. Total RNA was extracted from five sections of 5-micron thick paraffin block using QIAamp RNA Mini Kit (Qiagen, Germany). The RNA was quantitated using nanodrop.

VEGF-C (NM_005429) and housekeeping G6PDH (NM_000402) gene specific forward (f) and reverse (r) primers were designed to bind to two different exons using specific primer analysis software (Amplify 1.2) as follows. 

*VEGF-C*. (f) 5′-CACGAGCTACCTCAGCAAGA-3′, (r) 5′-GCTGCCTGACACTGTGGTA-3′ and the house keeping gene* G6PDH*. (f) 5′-TGGAGAATGAGAGGTGGGATG-3′; (r) 5′-GAGCTTCACGTTCTTGTATCTGT-3′.

Total RNA was reverse-transcribed followed by real-time PCR according to manufacturer's instructions. Real-time quantitative PCR was performed using SYBR Green Universal Master Mix (2X) (Applied Biosystems, Warrington, WA1 4SR, UK) according to the manufacturer's protocol. Cycle conditions were as follows: 95°C for 10 min followed by 45 cycles (95°C denaturation for 15 s, 60°C annealing for 1 min, and 72°C extension for 1 min), with a final incubation at 72°C for 5 min.

A relative quantification method (2^−ΔΔCT^ method was used, where Ct is cycle threshold) was chosen for quantitative analysis. The relative quantitation value of target was normalized to the endogenous control GAPDH (housekeeping) gene.

### 2.5. Statistical Analysis

The data obtained from the computer image analysis were tabulated and statistically analyzed. Data were presented as mean, standard deviation (SD) values, standard error of difference (SE), and 95% confidence interval (CI).

*One-way analysis of variance (ANOVA)* was used for the three groups (normal tissue, positive LN OSCC, and node-free OSCC) comparisons followed by* Turkey's post hoc test* for pair-wise comparison between the means when ANOVA test is significant.* Student's t-test* was used for the two groups' comparison (if any group gives a negative reaction).

*Pearson's correlation test* was used to study the correlation between the area percent of VEGF-C expression with LVD and LV surface area. When the value of the correlation coefficient lies around ±1, it points to a perfect degree of association between variables. When the value of the correlation coefficient goes towards 0, it means a weaker relationship between variables. The significance level was set at *p* ≤ 0.05.

## 3. Results

### 3.1. Detection of VEGF-C by Ordinary Light Microscope

**Figure 2:**
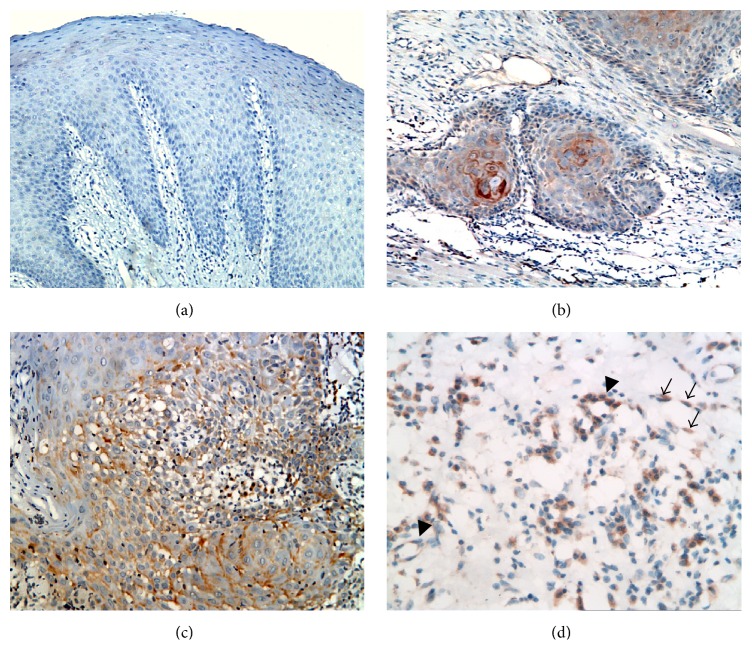
Photomicrographs showing negative immunoreaction of VEGF-C in normal mucosa ((a) ×200), weak positive VEGF-C expression in node-free OSCC ((b) ×200), and more diffuse positive expression in positive lymph nodes OSCC ((c) ×200), ((d) ×400). (d) shows VEGF-C expression by cancer associated fibroblasts (arrows) and blood endothelial cells (arrow heads).

The positive immunoreaction of VEGF-C was detected as a brownish color in surface epithelium, in the invading epithelial masses and in the tumor-associated stromal cells including fibroblasts and endothelial and inflammatory cells. It appeared as a granular, diffuse, or perinuclear reaction in the cytoplasm, while the nuclei negatively reacted (Figure 2).

In* normal mucosa*, all cases showed negative immunoreaction with VEGF-C.

In* node-free OSCC*, two cases did not express the VEGF-C neither in the lesional tissues nor in the adjacent marginal tissue. In the rest of cases, at least a localized area of positive expression was detected in cancer cells, stromal cells, or both, while the rest of lesion showed negative reaction.

In seven cases, a weak cytoplasmic immunoreaction was seen in cancer cells only. In two cases, a positive reaction was localized in stromal cells while the epithelial cancerous cells negatively reacted.

In all cases of* metastasizing OSCC*, a stronger and more diffuse expression was detected in both invading epithelial nests, associated stroma, and nearby dysplastic surface epithelium.

However, four cases showed the expression in invading masses, while the surface epithelium was negatively reacted.

In three cases, the expression of VEGF-C was restricted to the invasive front of epithelial cell nests.

In two cases, the expression was limited to the cancer associated stromal cells only, with strong expression in those surrounding epithelial nests.

### 3.2. Assessment of VEGF-C by Computer Image Analyzer

Generally, greater mean area percent of VEGF-C immunoexpression by the whole tumor tissue, epithelial component, and cancer associated stroma was recorded in the positive lymph node OSCC, compared to the negative lymph node OSCC.

A greater mean optical density of immunoexpression was also recorded in the positive lymph node OSCC, compared to the negative lymph node OSCC (Table 2).

### 3.3. Assessment of VEGF-C Gene Expression

A greater fold change compared to control was recorded in the positive lymph node OSCC (4.486 ± 1.86), compared to the negative lymph node OSCC (2.239 ± 0.785). Unpaired *t*-test revealed that the difference was statistically significant (*p* = 0.0002^*∗*^) (Table 2).

### 3.4. Detection of D2-40 Positive Lymphatic Vessels

**Figure 3:**
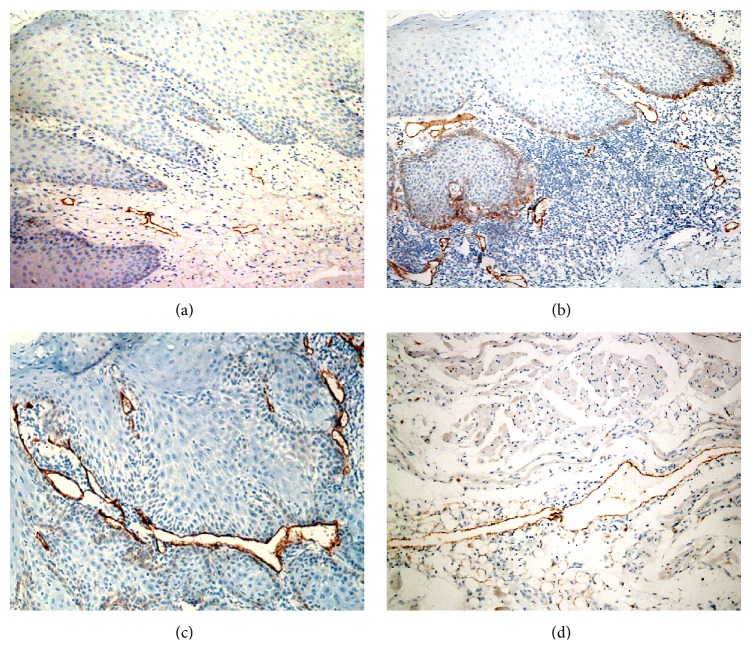
Photomicrographs of D2-40 stained sections showing few and superficially located LVs in normal mucosa ((a) ×200), more numerous LVs in node-free OSCC ((b) 200), and more dilated and deeply located LVs in positive lymph nodes OSCC ((c) ×200), ((d) ×100).

The positive immunoreaction of D2-40 was detected as a granular cytoplasmic expression in lymphatic endothelial cells, while blood endothelial cells negatively reacted (Figure 3).

*In normal mucosa,* lymphatic vessels were restricted to the subepithelial connective tissue.

In* node-free OSCC,* lymphatic vessels were more numerous and larger in size compared to those observed in normal mucosa and were detected superficially in the subepithelial connective tissue and deep in between malignant epithelial cell nests.

Subepithelial lymphatic vessels and those at the periphery of the lesion were dilated, while intratumoral vessels (in between epithelial cell nests) were compressed in between the invasive epithelial masses.

In* positive node OSCC,* lymphatic vessels were more enlarged and dilated (wider lumina) than those of normal mucosa and node-free OSCCs.

They were detected superficially in the subepithelial connective tissue, in between malignant epithelial masses, and deeply in between muscle fibers, in association with cancer cell nests. However, some lymphatic vessels were compressed, with hardly seen lumina, in between the invasive epithelial masses.

In six cases, no lymphatic vessels could be seen in between malignant epithelial cell nests.

### 3.5. Assessment of D2-40 Positive Lymphatic Vessels

#### 3.5.1. Lymphatic Vessel Density

The greatest mean LVD was recorded in the negative LN OSCC, whereas the lowest value was recorded in the normal mucosa group (Table 3).

#### 3.5.2. Lymphatic Vessel Surface Area

The greatest mean LV surface area (*μ*m^2^) was recorded in the positive LN OSCC, whereas the lowest value was recorded in the normal group (Table 3).

#### 3.5.3. Lymphatic Vessel Roundness

A value of 1.0 indicates a perfect circle. The greatest deviation from LV roundness was recorded in the negative group, whereas the lowest value was recorded in the positive group indicating that lymphatic vessels in positive node group were more close to roundness in comparison to the normal mucosa and negative node OSCC (Table 3).

### 3.6. Correlation between Area Percent of VEGF-C Expression and LVD and LV Surface Area

#### 3.6.1. Correlation of VEGF-C and LVD

The value of *R* is −0.67. This is a moderate negative correlation. The value of *R*^2^, the coefficient of determination, is 0.4482 (*p* value = 0.05).

#### 3.6.2. Correlation of VEGF-C and LV Surface Area

The value of *R* is 0.69. This is a moderate positive correlation. The value of *R*^2^, the coefficient of determination, is 0.4789 (*p* value = 0.05).

## 4. Discussion

In the present study, we investigated OSCC for the expression of VEGF-C at the gene and protein levels, in an attempt to examine the relation of such expression to the tumor lymphangiogenic activity, as a promoter mechanism for tumor progression.

The results of this study showed that normal oral mucosa was immunonegative to VEGF-C antibody. This is consistent with the fact that lymphangiogenesis occurs only during embryogenesis and is largely absent under normal physiologic conditions [11].

Furthermore, while the expression of VEGF-C was absent in normal mucosa, it was detected in 97% of OSCC cases. This can suggest that VEGF-C can be used as a diagnostic marker for malignant transformation of oral mucosa.

In consistency, VEGF-C was reported to be expressed in many human cancers but was not detected in their normal tissue counterparts [23, 25]. This result indicates that VEGF-C may be an attractive target in anticancer therapy that can be directed against cancer cells without harming normal tissue.

The present results showed also that both epithelial and connective tissue cells, in OSCCs, showed a cytoplasmic VEGF-C immunoreaction. Among positively reacting cancer associated stromal cells, VEGF-C was strongly expressed by fibroblasts, endothelial cells, and chronic inflammatory cells.

In consistency, it was previously reported that the expression of VEGF-C by malignant epithelial cells and cancer associated leukocytes differentiates the cancer associated lymphangiogenesis from that occurring during embryogenesis, in which the secretion of lymphangiogenic factors is restricted to fibroblasts and blood vessel cells [26].

The epithelial expression of VEGF-C was greater in positive node OSCC, compared to node-free cases. It was also noticed that some cases showed VEGF-C expression throughout all layers of invading masses, while the surface epithelium negatively reacted. In other cases, the expression of VEGF-C was observed in the invasive front of epithelial cell nests, while the central cells did not show any reaction.

Similarly, Sugiura et al. [21] and Yanase et al. [23] observed the more intense immunoexpression of VEGF-C in invasive front areas of the tumors, particularly in the positive node OSCC, and suggested a role for VEGF-C in facilitating the invasiveness of cancer cells. Sugiura et al. [21] attributed this increase in VEGF-C expression in invasive front to its ability to induce the production of urokinase by cancer cells, which accelerates plasmin-mediated matrix degradation and enables the cancer cells to grow and invade the surrounding tissue.

The results of the current study showed also that VEGF-C was expressed by OSCC associated stromal cells. It was also significantly higher in the node positive tumors, compared to node-free OSCCs. Some of the positively reacting stromal cells were observed around and in close contact with invading epithelial cell nests suggesting the interaction between cancer cells and associated stromal cells that enhances and facilitates cancer progression and metastasis.

Our results agree with the well documented role of tumor-associated stroma in promoting cancer metastasis. In fact, tumor-associated stromal cells, unlike cancer cells, are genetically more stable [27]. Therefore, targeting stromal cells expressing VEGF-C in OSCC may be an effective therapeutic strategy that can reduce metastasis of oral cancer and improve patient's prognosis.

In the present study, some blood endothelial cells expressed VEGF-C indicating their function as a source of this lymphangiogenic factor, that in turn stimulates the proliferation and sprouting of endothelial cells (paracrine mechanism). It was found that VEGF-C has the affinity to bind to VEGFR-2 and VEGFR-3, located on blood and lymphatic endothelial cells, respectively [28], leading to a dramatic change in endothelial cell morphology, actin reorganization, membrane ruffles, and lamellipodium extension [29] and inducing the formation of tip cells at the leading front of growing vessels [30].

In addition, VEGF-C also stimulates endothelial cells to secrete a number of chemokines that regulate the aggregation of leukocytes in the tumor-associated stroma [31].

In the present study, VEGF-C was also expressed by different types of inflammatory cells mainly macrophages. These cells were found to produce interleukin-6 (IL-6) that upregulates VEGF-C expression [32] inducing further lymphatic vessels growth and permeability [31]. This is beside their ability to secrete chemokines, reactive oxygen species, and matrix metalloproteinases (MMPs) that modulate angiogenesis, cell proliferation, tumor growth, and invasion [33].

Therefore, VEGF-C plays an important role in the remodeling of the extracellular matrix not only through the release of urokinase by cancer cells [21] but also by MMPs released by leukocytes recruited by VEGF-C [33].

In addition, VEGF-C has been identified as prolymphangiogenic factor that activates VEGFR-3 expressed on lymphatic endothelium. This has been supported by the observation in mouse model that blocking signaling via VEGFR-3, using fusion protein that act as a trap for VEGF-C, reduced tumor lymphangiogenesis and lymphatic spread [28].

Accordingly, the frequent expression of VEGF-C in OSCC, detected in this study, suggested that lymphangiogenic activity might take place in these tumors.

Therefore, in this study, we examined the relationship between VEGF-C expression, lymphangiogenic activity, and lymph node metastasis in OSCC.

The results showed a direct correlation between the expression level of VEGF-C and lymph node metastasis. This is consistent with previous studies on human tumors of diverse tissue origins showing elevated VEGF-C that significantly correlated with lymphatic spread and node metastasis [9, 21, 22, 34–36].

In addition, the results of the present study showed that lymphatic vessels in OSCC were more numerous, larger in size, and more deeply located than those seen in normal mucosa. Furthermore, larger, more elongated, and dilated lymphatic vessels with wider lumina were associated with increased level of VEGF-C observed in positive lymph node OSCC, compared to negative node group.

Similar results have been observed by Sugiura et al. [21] and Ran et al. [37] who pointed to the role of VEGF-C in increasing the size of lymphatic vessels and their permeability to tumor cells, beside its lymphangiogenic property, facilitating the intravasation and dissemination of cancer cells.

On the other hand, the present study showed that a greater number of lymphatic vessels (LVD) were detected in negative node OSCC as compared to positive node group. This result is consistent with Watanabe et al.'s [19] study that suggested that lymphatic vessels may be damaged, with time, by proliferating malignant cells and concluded that LVD does not necessarily indicate the risk of cervical lymph node metastasis.

Therefore, during carcinogenesis, the lymphangiogenic factor VEGF-C is overexpressed by both malignant epithelial cells and the associated stromal cells stimulating the formation of new lymphatic vessels. With the progression of cancer, and despite the continuous production of VEGF-C, lymphatic vessels may persist and can be detected by lymphatic markers or may be damaged by proliferating malignant cells leading to reduction in LVD. In addition, VEGF-C increases the size and permeability of the remaining lymphatic vessels promoting the spread of cancer cells to the regional lymph node.

Using PCR, small amounts of VEGF-C mRNA could be detected in the specimens of normal mucosa, and the fold change in OSCC could be calculated. Results obtained by PCR have superior sensitivity, since low amount of VEGF-C protein can escape detection by immunohistochemistry.

The level of gene expression was higher in metastasizing cases of OSCC than in node-free group which is consistent with Shintani et al. [9] and Cai et al. [36] results and with the immunohistochemical findings of the present study. Taken together, all these findings highlight the role of VEGF-C in invasive and metastasizing stages of OSCC.

## 5. Conclusion

VEGF-C is actively involved in the invasion and metastasis of OSCC via inducing morphological changes in LV. VEGF-C can be used as a diagnostic and a prognostic marker. It provides a promising target for anticancer therapy.

## Figures and Tables

**Table 1:** Clinical data and histological grades of the studied cases.

Clinical features	Positive LN OSCC	Negative LN OSCC
*Gender*		
Male	24 (66.7%)	20 (55.6%)
Female	12 (33.3%)	16 (44.4%)

*Age*	56–68 (Mean = 59.42)	50–75 (Mean = 59.69)

*Site *		
Tongue	15 (41.7%)	4 (11.1%)
Lip	4 (11.1%)	—
Buccal mucosa	4 (11.1%)	—
Gingiva	4 (11.1%)	12 (33.4%)
Alveolar mucosa	8 (22.2%)	8 (22.2%)
Palate	1 (2.8%)	4 (11.1%)
Retromolar area	—	8 (22.2%)

*LN palpation*	34/36 (94.4%)	6/36 (16.7%)

*Histological grades*		
Well	14 (38.9%)	20 (55.6%)
Moderate	18 (50%)	13 (36.1%)
Poor	4 (11.1%)	3 (8.3%)

**Table 2:** Assessment of VEGF-C expression.

	Positive LN OSCC		Negative LN OSCC		*p* value	95% CI	SE of difference
	Mean	SD	Mean	SD			
(I) IHC (area percent)	22.23	2.228	8.63	1.818	0.00001	12.55 to 14.65	0.53
(I.1) Total expression							
(I.2) Epithelial expression	10.93	1.53	4.05	1.85	0.0002	6.00 to 7.76	0.44
(I.3) Stromal expression	4.42	1.54	2.55	0.61	0.0356	1.26 to 2.48	0.30
(II) IHC (optical density)	57.1	0.339	43.5	2.66	0.001	12.62 to 14.58	0.49
(III) RT-PCR	4.486	1.86	2.239	0.785	0.0002	1.179 to 3.31	0.52

**Table 3:** Assessment of LV density, surface area, and roundness.

	Normal mucosa (*n* = 6)		Positive LN OSCC (*n* = 30)		Negative LN OSCC (*n* = 30)		*p* value	Between normal and positive LN OSCC		Between normal and negative LN OSCC		Between positive and negative LN OSCC	
	Mean	SD	Mean	SD	Mean	SD		95% CI	SE	95% CI	SE	95% CI	SE
(I) LVD	5.5	0.58	6.88	0.95	8.33	0.76	0.0001	−2.20 to −0.56	0.4	−3.5 to −2.16	0.33	−1.89 to −1.01	0.22
(II) LV area (*μ*m^2^)	242723	81526	337929	97914	289621	59013	0.045	−182164.66 to −8247.34	42789.49	−104002.39 to 10206.39	28099.19	6527.4 to 90088.6	20872.38
(III) LV roundness	2	0.4	1.8	0.5	2.1	0.6	0.026	−0.24 to 0.64	0.22	−0.623 to 0.423	0.26	−0.59 to −0.02	0.14
